# Data for default network reduced functional connectivity in meditators, negatively correlated with meditation expertise

**DOI:** 10.1016/j.dib.2016.07.015

**Published:** 2016-07-15

**Authors:** Aviva Berkovich-Ohana, Michal Harel, Avital Hahamy, Amos Arieli, Rafael Malach

**Affiliations:** a Department of Neurobiology, Weizmann Institute of Science, Rehovot 76100, Israel; b Faculty of Education, The Edmond J. Safra Brain Research Center for the Study of Learning Disabilities, University of Haifa, Haifa, Israel

## Abstract

FMRI data described here was recorded during resting-state in Mindfulness Meditators (MM) and control participants (see “Task-induced activity and resting-state fluctuations undergo similar alterations in visual and DMN areas of long-term meditators” Berkovich-Ohana et al. (2016) [1] for details). MM participants were also scanned during meditation. Analyses focused on functional connectivity within and between the default mode network (DMN) and visual network (Vis). Here we show data demonstrating that: 1) Functional connectivity within the DMN and the Visual networks were higher in the control group than in the meditators; 2) Data show an increase for the functional connectivity between the DMN and the Visual networks in the meditators compared to controls; 3) Data demonstrate that functional connectivity both within and between networks reduces during meditation, compared to the resting-state; and 4) A significant negative correlation was found between DMN functional connectivity and meditation expertise. The reader is referred to Berkovich-Ohana et al. (2016) [1] for further interpretation and discussion.

**Specifications Table**

**Table:** 

**Subject area**	Psychology
**More specific subject area**	Cognitive Neuroscience, fMRI
**Type of data**	Figure
**How data was acquired**	fMRI scanning (3 T Trio Magnetom Siemens scanner).
**Data format**	Analyzed
**Experimental factors**	For each subject and ROI the mean time course was extracted and averaged bilaterally. Functional connectivity was calculated using Pearson correlation.
**Experimental features**	7 min of resting-state and meditation activity
**Data source location**	The Weizmann Institute of Science, Rehovot, Israel
**Data accessibility**	Data is within this article

**Value of the data**

• Data can be used for comparison to previous reports of rest functional connectivity in meditators compared to controls, both in the DMN [2], [3] and in visual/sensory regions.
• Previous reports reported increased functional connectivity between the DMN and sensory (including visual) networks in meditators compared to controls during meditation [4], [5]. The current data enables a comparison with these reports, by reporting a similar effect during rest.
• The data can be used to examine meditative training effects by comparing DMN functional connectivity and meditation expertise, and can be useful in comparison with similar reports [6].

## 1 Data

**Fig. 1:**
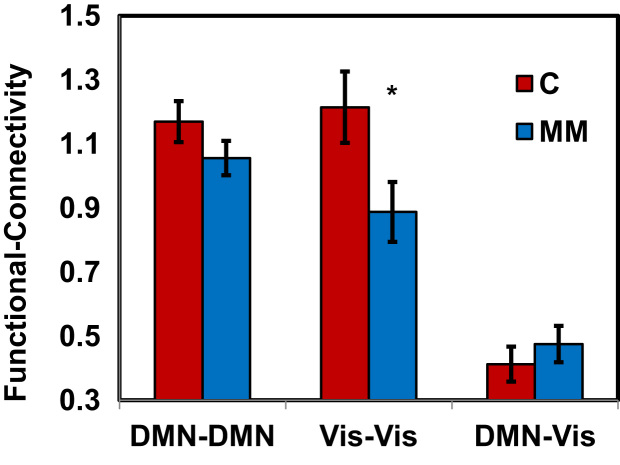
The significant Group×Network_FC interaction, showing reduced resting-state functional connectivity values for the mindfulness meditation (MM) group compared to the control (C) group. *p<.05.

**Fig. 2:**
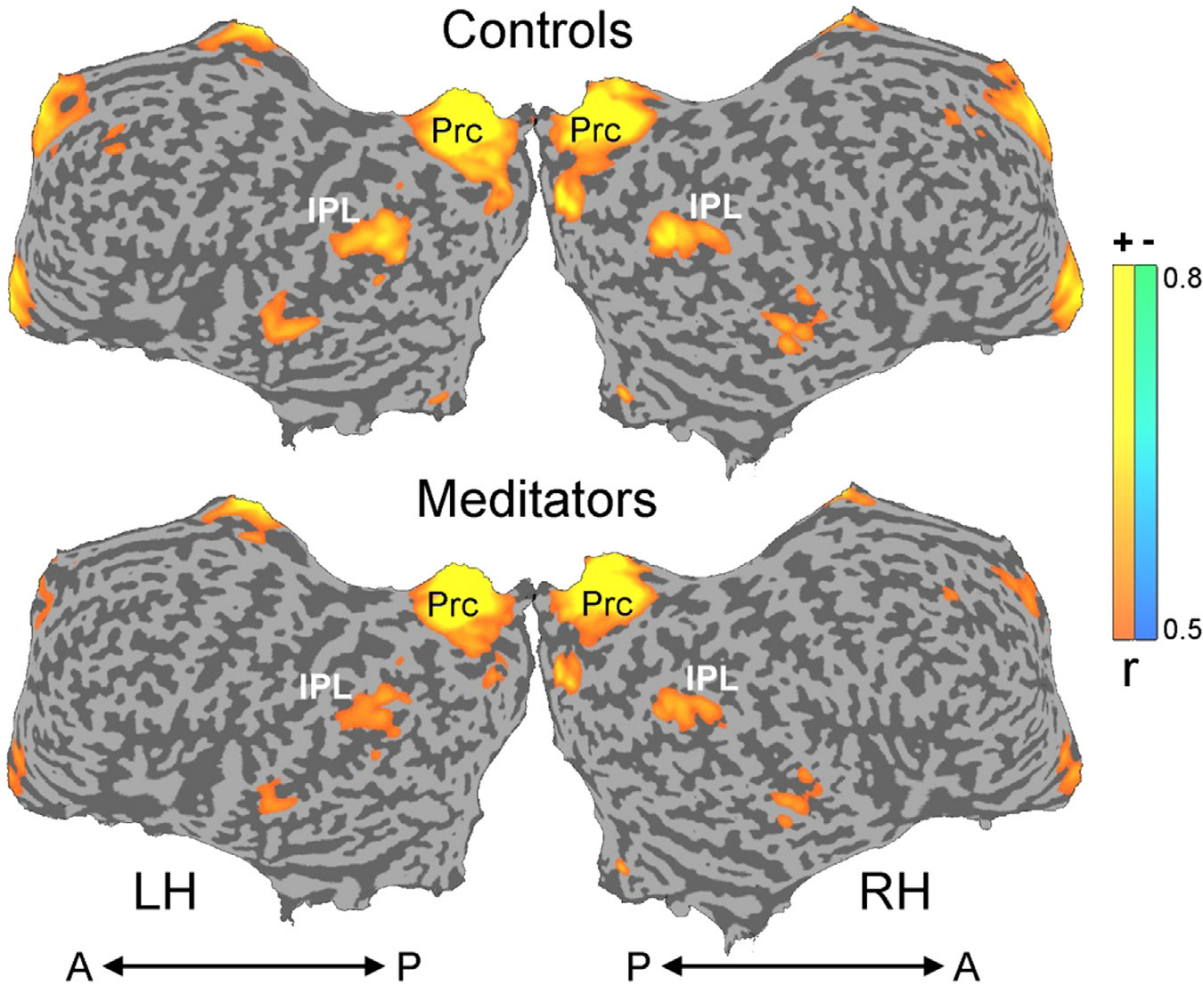
A qualitative demonstration of resting state functional connectivity (unfolded brain view), derived from bilateral Prc as a seed. Colour bar indicates positive correlations in yellow, and negative correlations in blue. LH, left hemisphere; RH, right hemisphere; Prc – precuneus; IPL – inferior parietal lobule; A, anterior; P, posterior.

**Fig. 3:**
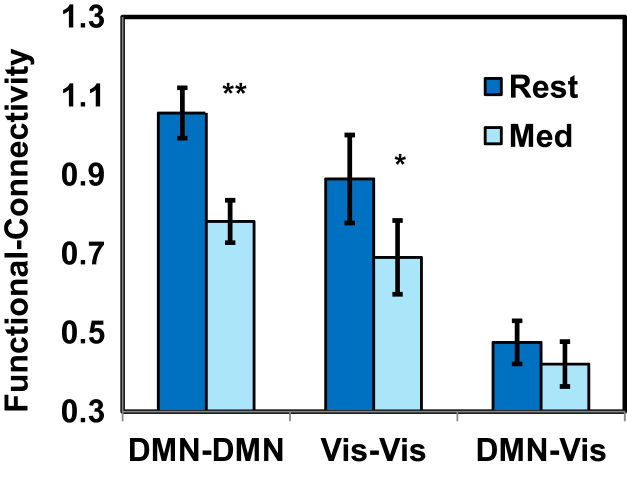
The significant Condition×Network_FC interaction, showing reduced functional connectivity values during meditation compared to resting-state for the MM group. *p<.05; **p<.001.

The fMRI data was derived from two groups of participants during resting state and only one of the groups during meditation. See Fig. 1, Fig. 2, Fig. 3.

## 2 Experimental design, materials and methods

fMRI data described here was recorded with 3 T Trio Magnetom Siemens scanner, during resting state (7 min.) in 18 Mindfulness meditators (MM, age 42.3±9.9 years, 6 female), and 18 meditation-naïve control participants (age 42.5±10.4 years, 5 female). MM participants were also recorded during meditation (7 min., focusing attention on breath and body sensations). For full details of fMRI data recording, experimental design, and preprocessing, see [1].

Default mode network (DMN) and visual network (Vis) bilateral regions of interest (ROIs) were identified using a localizer task [1], and included two ROIs for DMN: the Precuneus (Prc) and Inferior parietal Lobule (IPL), and two ROIs for visual: lateral occipital (LO) and posterior fusiform (pFs) (For ROI definition, see [1]). Homologue bilateral ROIs were collapsed together, yielding two DMN ROIs (bilateral_IPL and bilateral_Prc) and two visual ROIs (bilateral_LO and bilateral_pFs) time courses.

Functional connectivity was calculated using Pearson correlation across the time courses, for each network and their combination (DMN-DMN, Vis–Vis, and DMN-Vis). These values were Fisher Z-transformed for normalisation, and then subjected to a 2-way ANOVA (Group×Networks_FC).

## 3 Comparing resting-state functional connectivity between groups

We found a main effect to the Networks_FC [*F*(2, 66)=53.94, *p*<.001], where the DMN-DMN connectivity values were the highest, and the DMN-Vis connectivity values were the lowest. Importantly, we found a significant Group×Networks_FC interaction [*F*(2, 66)=3.78, *p*<.05]: while the functional connectivity within the DMN and the Vis networks were higher in the control group than in the meditators [post-hoc for the Vis–Vis *t*-test: *t*=4.87, *p*<.05], the situation was reversed for the functional connectivity between the networks, i.e. DMN-Vis connectivity was higher in the meditators compared to the control group (Fig. 1). The data is demonstrated visually in Fig. 2.

## 4 Comparing resting-state and meditation functional connectivity

We tested FC difference between rest and meditation in the MM group, using a 2-way ANOVA (Condition×Networks_FC), and found a main effect for Condition [[*F*(1, 32)=12.86, *p*<.001], stemming from a significant reduction in FC both in the DMN and the Vis network during meditation compared to rest [post-hoc paired t-test, *t*=5.53, *p*<.001; and *t*=2.46, *p*<.05, respectively] (Fig. 3).

## 5 Relationship between functional connectivity and meditation expertise

A significant negative correlation was found between DMN-DMN connectivity and MM expertise (years) (*r*= −.340; *p*<.05).
